# Chemokine CXCL12 Activates CXC Receptor 4 Metastasis Signaling Through the Upregulation of a CXCL12/CXCR4/MDMX (MDM4) Axis

**DOI:** 10.3390/cancers16244194

**Published:** 2024-12-16

**Authors:** Rusia Lee, Viola Ellison, Dominique Forbes, Chong Gao, Diana Katanov, Alexandra Kern, Fayola Levine, Pam Leybengrub, Olorunseun Ogunwobi, Gu Xiao, Zhaohui Feng, Jill Bargonetti

**Affiliations:** 1 Department of Biological Sciences, Hunter College, City University of New York, Belfer Building, New York, NY 10021, USA; rlee2@gradcenter.cuny.edu (R.L.); ve226@hunter.cuny.edu (V.E.);; 2 Biology and Biochemistry Programs, The Graduate Center, City University of New York, New York, NY 10016, USA; 3 Department of Cell and Developmental Biology, Weill Cornell Medical College, New York, NY 10021, USA; 4 Department of Radiation Oncology, Rutgers Cancer Institute, Rutgers, State University of New Jersey, New Brunswick, NJ 08901, USA; fengzh@cinj.rutgers.edu

**Keywords:** triple-negative breast cancer (TNBC), metastasis, circulating tumor cells (CTCs), MDM2, MDMX, CXCR4, PI3K/AKT signaling, tumor microenvironment (TME), chemokine signaling

## Abstract

Background: The metastasis-promoting G-protein-coupled receptor CXC Receptor 4 (CXCR4) is activated by the chemokine CXCL12, also known as stromal cell-derived factor 1 (SDF-1). The CXCL12/CXCR4 pathway in cancer promotes metastasis but the molecular details of how this pathway cross-talks with oncogenes are understudied. An oncogene pathway known to promote breast cancer metastasis in MDA-MB-231 xenografts is that of Mouse Double Minute 2 and 4 (MDM2 and MDM4, also known as MDMX). MDM2 and MDMX promote circulating tumor cell (CTC) formation and metastasis, and positively correlate with a high expression of CXCR4. Interestingly, this MDMX-associated upregulation of CXCR4 is only observed in cells grown in the tumor microenvironment (TME), but not in MDA-MB-231 cells grown in a tissue culture dish. This suggested a cross-talk signaling factor from the TME which was predicted to be CXCL12 and, as such, we asked if the exogenous addition of the cell non-autonomous CXCL12 ligand would recapitulate the MDMX-dependent upregulation of CXCR4. Methods: We used MDA-MB-231 cells and isolated CTCs, with and without MDMX knockdown, plus the exogenous addition of CXCL12 to determine if MDMX-dependent upregulation of CXCR4 could be recapitulated outside of the TME context. We added exogenous CXCL12 to the culture medium used for growth of MDA-MB-231 cells and isogenic cell lines engineered for MDM2 or MDMX depletion. We carried out immunoblotting, and quantitative RT-PCR to compare the expression of CXCR4, MDM2, MDMX, and AKT activation. We carried out Boyden chamber and wound healing assays to assess the influence of MDMX and CXCL12 on the cells’ migration capacity. Results: The addition of the CXCL12 chemokine to the medium increased the CXCR4 cellular protein level and activated the PI3K/AKT signaling pathway. Surprisingly, we observed that the addition of CXCL12 mediated the upregulation of MDM2 and MDMX at the protein, but not at the mRNA, level. A reduction in MDMX, but not MDM2, diminished both the CXCL12-mediated CXCR4 and MDM2 upregulation. Moreover, a reduction in both MDM2 and MDMX hindered the ability of the added CXCL12 to promote Boyden chamber-assessed cell migration. The upregulation of MDMX by CXCL12 was mediated, at least in part, by a step upstream of the proteasome pathway because CXCL12 did not increase protein stability after cycloheximide treatment, or when the proteasome pathway was blocked. Conclusions: These data demonstrate a positive feed-forward activation loop between the CXCL12/CXCR4 pathway and the MDM2/MDMX pathway. As such, MDMX expression in tumor cells may be upregulated in the primary tumor microenvironment by CXCL12 expression. Furthermore, CXCL12/CXCR4 metastatic signaling may be upregulated by the MDM2/MDMX axis. Our findings highlight a novel positive regulatory loop between CXCL12/CXCR4 signaling and MDMX to promote metastasis.

## Simple Summary

Breast cancer metastasis is promoted by a number of factors, including signaling from the stromal cell-derived factor 1 (SDF-1) to the CXC Receptor 4 (CXCR4), and mutations in the *TP53* gene in combination with the upregulation of Mouse Double Minute 2 and 4 (MDM2 and MDM4). However, the signal transduction connection between these breast cancer metastases-promoting pathways has never been identified. In this study, we identified that SDF-1 not only signals the upregulation of CXCR4 as previously reported, but we also discovered new signal transduction connections. The addition of the SDF-1 chemokine to breast cancer cells upregulated MDM2 and MDM4. Furthermore, the knockdown of MDM4 inhibited the ability of SDF-1 to signal for CXCR4 upregulation and increased in vitro cell migration. As such, we identified an SDF-1/CXCR4/MDM2/MDM4 signal transduction axis that we speculate participates in driving breast cancer metastasis.

## 1. Introduction

Breast cancer metastasis is promoted by multiple factors including notable contributors such as C-X-C chemokine receptor 4 (CXCR4 also known as CD184), Mouse Double Minute 2 (MDM2), and Mouse Double Minute 4 (MDM4 also known as MDMX) [1,2,3,4]. Both MDM2 and MDMX promote the circulating tumor cells (CTCs) required for the dissemination of xenograft MDA-MB-231 human triple-negative breast cancer (TNBC) cells to travel to the lung to form metastases [4]. Additionally, CTCs that can form metastasis use the CXCR4-PI3K/AKT pathway to survive fluid sheer stress [5]. The stable knockdown of both MDM2 and MDMX in the MDA-MB-231 cells in a xenograft mouse model significantly decreased the number of CTCs but MDMX depletion is more effective at reducing CTCs and metastasis [4]. Moreover, the knockdown of MDMX in primary xenograft tumors in the tumor microenvironment (TME), but not in cell culture, correlates with a lower expression of the well-studied metastasis-promoting receptor CXCR4 [4]. Chemokine receptor CXCR4 is a highly conserved G-coupled protein receptor (GCPR) first discovered in 1996 as being required for HIV entry into CD4+ T cells [6] and later identified as being expressed in various cell types including hemopoietic stem cells, stromal fibroblast cells, and cancer cells [7,8].

CXCR4 mediates breast cancer metastasis to the lungs [9]. The chemokine CXCL12, also known as stromal cell-derived factor-1 (SDF-1), binds with high affinity to the CXCR4 receptor and functions to drive breast cancer progression [9]. The CXCL12-CXCR4 axis is important in promoting cell growth, proliferation, and migration (reviewed in [10]). A study comparing primary tumors derived from MDA-MB-231 xenograft mice to MDA-MB-231 parental cells grown in a cell culture showed that, with exogenous CXCL12, the primary tumor cells expressed more CXCR4 and activated PI3K/AKT signaling [8]. Importantly, the addition of CXCL12 increases chemotaxis and participates in a positive feedback loop to increase cancer cell expression of CXCR4 [11]. Furthermore, the CXCL12/CXCR4-AKT axis activates the downstream RhoA/ROCK2 pathway, which modulates cell invasion and tumor metastasis [12]. Moreover, the ectopic expression of CXCR4 in mammary epithelial cells enhances the expression of receptor tyrosine kinases and MDM2 [13]. Connections have been made between CXCR4 activity and MDM2 and MDMX, showing that they work together to promote metastasis [3,4,14], but no study has examined how CXCL12 contributes to the signaling axis. Because we previously recognized the dichotomy between the influence of MDMX expression correlating with high levels of CXCR4 in the TME, but not in cell culture, we asked if stromal-derived CXCL12 could be the missing link. Herein, we demonstrate that CXCL12 activates CXCR4 signaling through a pathway that is facilitated by the expression of MDMX.

## 2. Materials and Methods

### 2.1. Cell Lines

Cells were maintained in DMEM (Invitrogen, Waltham, MA, USA) medium supplemented with 10% fetal bovine serum (FBS, Gemini, West Sacramento, CA, USA), 50 U/mL penicillin, and 50 μg/mL streptomycin (Mediatech, Bedminster, NJ, USA) at 5% CO_2_ and 37 °C in a humidified incubator. All commercially available human cell lines were purchased from American Type Culture Collection (ATCC, Manassas, VA, USA). The details of these cell lines can be found on the ATCC website for human breast cancer cell lines MDA-MB-231, MDA-MB-468, MCF-7, and T47D. The isogenic derivatives of MDA-MB-231 were described and named previously and included the vector control MDA-231.mlp for the cells expressing the MLP vector and for those with constitutive shRNA knockdown against *mdm2* or *mdmx* they were named MDA-MB-231.sh*mdm2* and MDA-MB-231-shmdmx, respectively. The HCT116 *TP53* knockout cells were from a well characterized colorectal cancer line generously given to us by Bert Vogelstein [15]. Circulating tumor cell (CTC) lines were generated by collecting whole blood from xenograft mouse experiments as described previously [16] and authenticated by short tandem repeat testing. The CTC lines were derived from MDA-MB-231, MDA-MB- 231.sh*mdm2,* and MDA-MB-231.sh*mdmx* cell lines orthotopically injected into mice and were named accordingly as MDA-MB-231.mlp.CTC (A, B: individual mice), MDA- MB-231.mlp.sh*mdm2.*CTC and MDA-MB-231.mlp.sh*mdmx.*CTC. T47D cells with inducible *Mdm2* knockdown were established as previously described [17,18]

### 2.2. Plasmid Construction

pcDNA3-MDMX was constructed by PCR amplification of MDMX from MDA-MB-231 cells. Restriction enzymes HindIII and Kpn1 (New England Labs, Woburn, MA, USA) were used to excise pcDNA3 and MDMX to create overhangs for MDMX. Ligation was performed to insert MDMX into the pcDNA3 vector.

### 2.3. Wound Healing Assay

Cells were plated onto a six-well plate a day before the experiment. Images were taken of the cell confluency at time zero. Scratches were created using a 200 μL pipette tip. Cells were then rinsed three times with fresh medium. Wound closure was observed within the scrape line and photographed by phase contrast microscopy during a time course experiment. The wound area was measured and quantified by using NIS-Elements software version 4.20 (Nikon Instruments, Melville, NY, USA).

### 2.4. Chemotaxis Colorimetric Assay

A 24-well transwell system using cell culture inserts (BD Falcon Cell Culture Inserts with a pore size of 8 µm) was used to determine the migratory abilities of MDA-MB-231 and the knockdown cell lines. Then, 50,000 cells were resuspended in 1x DMEM media with no FBS at a total volume of 200 µL. CXCL12 at 50 ng/mL was added to the lower chamber in a 1x DMEM medium supplemented with 10% FBS at a total volume of 500 µL. Cells were left to migrate through the membrane pores of the insert over a 24 h period at 37 °C and 5% CO_2_ incubator. Inserts were washed with water, stained with crystal violet, washed, and left to dry. Experiments were carried out in duplicate per condition with three images taken per well. Images were measured using NIS-Elements software (Nikon Instruments, Melville, NY, USA) and cell counts were quantified by ImageJ analysis.

### 2.5. Cell Lysates

Cells were harvested by scraping cells from tissue culture plates and washing them with ice-cold PBS. Cells were pelleted down and lysed in CHAPS buffer containing 50 mM KPO pH 7.41, 1 mM DTT, 1 mM EDTA, 100 µM NaV, 50 mM NaF, 250 mM NaCl, 7 mM CHAPS, 10% glycerol, 10 mM beta-glycerol phosphate, 0.5 mM PMSF, 5.0 µg/mL leupeptin, and 1 pellet of protease inhibitor mix/10 mL. Cells were vortexed in lysis buffer for 30 min on ice and vortexed every 5 min followed by sonication until lysis was visible and clear to the eye and spun down at 13,000 rpm for 20 min. Supernatant was collected.

### 2.6. Immunoblotting

Samples were boiled at 70 °C for 10 min with a final concentration of 100 mM iodoacetamide (Sigma, MO, USA) were added to prevent formation of disulfide bridges. Samples were run on a 10% SDS-PAGE gel to separate the proteins. A nitrocellulose (GE Life Science, Marlborough, MA, USA) membrane was used in the electro transfer of proteins. The membrane was blocked with 5% non-fat milk in 1-timePBS/0.1% Tween-20 (PBST) was used, followed by 2 washes with 1-time PBST and incubation with the primary antibody overnight at 4 °C. Washes were performed 2-times with 1X PBST, and the membrane was incubated with secondary mouse or rabbit antibody, Cy-3 and Cy-5, respectively (GE Life Science), for 1 h at room temperature in a dark setting. Further washes were performed 3x with 1X PBST and 1x with 1x PBS to wash off the detergent.

### 2.7. Antibodies

MDM2 R&D Systems Cat no. AF1244 (1:2000) Rabbit in 1% milk PBST, MDMX, 53BP1 Cell Signaling (1:1000) Rabbit in 1% milk TBST, PARP-1 Proteintech (1:1000) Rabbit in TBST, GST Santa Cruz Technologies (1:2000) Mouse in 1% milk PBST, Ubiquitin Cell Signaling Rabbit Polyclonal Ab (1:10,000) in 5% BSA PBST, CXCR4 mouse antibody Proteintech (1:2000) Cat No. 60042-1-Ig in 1% milk PBST, phospho-AKT S473 Cell Signaling (1:1000) Cat No. 9217S Rabbit 5% BSA in TBST, and MDMX (Cat No. 17914-1-AP; Proteintech, Rosemont, IL, USA).

### 2.8. RNA Isolation and Quantitative Real-Time Polymerase Chain Reaction

RNA was extracted from MDA-MB-231 or MDA-MB-231.mlp.CTC cells with appropriate cell treatments using QIAshredder columns and the RNeasy Mini Kit (Qiagen, Hilden, Germany) following the manufacturer’s protocol. Complementary DNA (cDNA) synthesis was carried out using the High-Capacity cDNA Archive Kit reagents (Applied Biosystems, Foster City, CA, USA). RT Master Mix and RNA were mixed and incubated at 25 °C for 10 min and then at 37 °C for 2 h. The amplification of gene transcripts was performed by qPCR with primer probes from Applied Biosystems for *MDM2* (Hs01069930_m1), *MDMX* (Hs00910358_s1), *CXCR4* (Hs00607978_s1), and *GAPDH* (Ha02758991_g1). Primers were combined with 150 ng of cDNA and TaqMan Universal Master Mix (Applied Biosystems, Waltham, MA, USA), and reactions were carried out using the standard program in the QuantStudio 7 sequence detection system (Applied Biosystems, Waltham, MA, USA). cDNA (25 ng) from samples was used (Thermo Fisher Scientific, Waltham, MA, USA).

### 2.9. Cell Treatments

Cells were seeded onto 10 cm plates and grown overnight to reach a confluency of about 70–80%. Cells were treated with CXCL12/SDF-1 α, Human Recombinant Animal-free (Millipore; Cat No. GF344). Cells were treated with cycloheximide (Sigma Aldrich; Cat No. C1988-1G) at a final concentration of 50 µg/mL dissolved in DMSO for 40 or 80 min or with DMSO as a vehicle. Cells were treated with MG132 final concentration at 10 µM in DMSO or with DMSO as a vehicle for 4 h.

### 2.10. Cell Transfections

Plasmids were transfected into HCT116 p53-/- using Nucleofection (Thermo Fisher Neon Transfection System; Cat No. MPK5000). Cells were grown in McCoy’s 5A media + 10% FBS with penicillin and streptomycin. Then, 3 µg of plasmid DNA and 1 × 10^6^ cells per condition were electroporated in Buffer R (Thermofisher) to a total volume of 120 µL and plated on 6 cm plates with McCoy’s 5A media + 10% FBS without antibiotics. Cells transfected with CMV-eGFP were verified for GFP expression 24 h after transfection and cells were harvested.

## 3. Results

### 3.1. Exogenous Addition of CXCL12 to MDA-MB-231 Cells Activates PI3K/AKT Signaling and Upregulates MDMX or MDM2 Protein Levels

**Figure 1:**
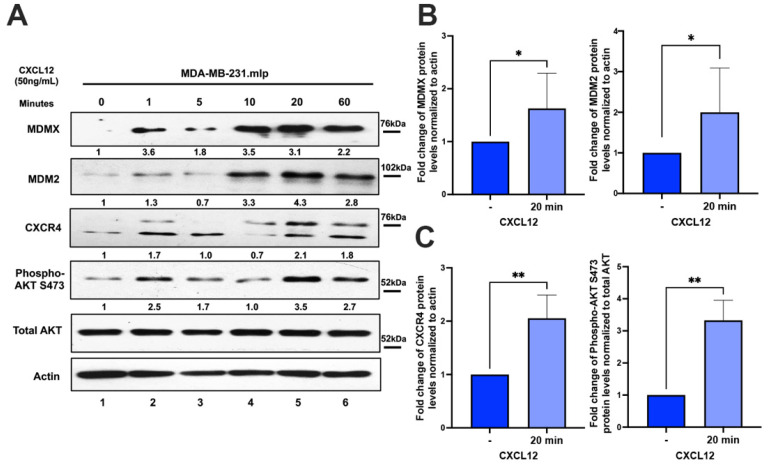
Chemokine CXCL12 addition to cell culture increases CXCR4, activates PI3K/AKT, and MDMX and MDM2 protein levels. (**A**) Panels show immunoblot analysis for MDMX, MDM2, CXCR4, and phospho-AKT S473, total AKT, and actin in MDA-MB-231.mlp cells after treatment for up to 60 min with CXCL12 at 50 ng/mL for 0, 1, 5, 10, 20, and 60 min, lanes 1–6. Total AKT and actin were used as loading controls. The proteins were derived from the same samples run on different gels/membranes at the same time and their molecular weights are shown. (**B**) Untreated cells and those following the 20 min treatments were compared for the MDMX and MDM2 protein levels evaluated (using actin as a normalizer control for loading) using ImageJ and graphs were created with Prism 10 software, with the untreated value set as 1 and the ratio reported for CXCL12-treated samples (20 min) used to report the fold change. (**C**) Untreated and 20 min treatments were compared for CXCR4 and phospho-AKT S473 protein levels quantified via ImageJ relative to total AKT as a loading control with the untreated value set as 1 and the ratio reported for CXCL12-treated samples (20 min) used to report the fold change. Images were analyzed using ImageJ and graphs were created with Prism software. Error bars represent SD. * *p* < 0.05, ** *p* < 0.01, NS = non-significant (N = 4 biological replicates).

The high CXCR4 expression in the TNBC primary xenograft tumors, but not in the cell culture, caused us to ask if the CXCR4 stromal-expressed ligand, CXCL12, was responsible for the MDMX-associated upregulation of CXCR4 in the tumor [4]. To investigate if factors in the tumor microenvironment mediate the MDMX-dependent upregulation of CXCR4, we explored if adding the stromal-derived extracellular signaling chemokine CXCL12 to cells grown in a culture would recapitulate the results observed in the xenograft tumors. CXCL12 is the exclusive ligand for CXCR4 [19,20], and has been shown to produce a signal in MDA-MB-231 cells to activate the CXCR4 receptor [2]. We added exogenous CXCL12 at 50 ng/mL to MDA-MB-231 cells in a cell culture and monitored the signal activation over time (Figure 1A). Signaling following the addition of CXCL12 has previously been shown to activate the upregulation of CXCR4, and is rapid and transient; therefore, we monitored time points at 0, 1, 5, 10, 20, and 60 min post the addition of 50 ng/mL CXCL12 for phospho-AKT (S473) (as previously published for signaling to activate AKT and upregulate CXCR4) [8,11,21] (see Figure 1). We observed the previously described activation of AKT and the upregulation of CXCR4 at the 20 and 60 min time points (Figure 1A, CXCR4 and Phospho AKT S473 panels, lanes 5 and 6). As early as 1 min post the addition of CXCL12, we observed a significant increase in MDMX and MDM2 protein levels (Figure 1A, lanes 1–6). This is consistent with previous reports on the kinetic analysis of signaling to the chemokine receptor CXCR4 [22]. Moreover, the concentration used is only slightly above what has been reported for CXCL12 serum levels (and the levels near tumors have never been reported) [23]. This demonstrated that the CXCL12 extracellular stromal-derived signal in the TME not only upregulates CXCR4, but can also upregulate MDM2 and MDMX (Figure 1). By examining cellular outcomes following the addition of exogenous CXCL12, we uncovered a cross-talk with CXCL12 to increase both MDM2 and MDMX.

Because CXCL12 signaling is rapid and transient, we quantified the upregulation of MDMX and MDM2 at 20 min post CXCL12 addition from four biological replicates and observed this to be highly reproducible (Figure 1B). Moreover, we observed an increase in the higher molecular weight form, and quantified this species for the 20 min post treatment value (Figure 1C) [20,24,25]. Activation of the PI3K/AKT signaling pathway was identified by increased phospho-AKT S473 (Figure 1C). We also tested the influence of CXCL12 on two other mutant p53-expressing breast cancer cell lines, MDA-MB-468 and T47D, and the wild-type p53-expressing breast cancer cell line MCF7 (Figure S1). While we observed the activation of phospho-AKT S473, we only found it to yield a non-statistically significant increase in CXCR4, MDM2, and MDMX (Figure S1). The cell context, and TME, has been demonstrated to have many alternative outcomes for mutant p53 and MDM2/MDMX signaling [26,27]. Because in MDA-MB-231 cells the addition of CXCL12 dramatically upregulated MDMX and MDM2 protein levels (Figure 1B), we asked if *MDM2* and *MDMX* mRNA expression increased and found that it did not (Figure S2).

### 3.2. MDMX Expression in MDA-MB-231 Cells Is Needed for CXCL12 Signaling to Upregulate CXCR4 and AKT Activation

We previously found that CXCR4 is highly expressed in xenograft primary tumors in an MDMX-dependent manner [4], so, herein, we asked if either MDM2 or MDMX was required for the CXCL12-mediated upregulation of CXCR4. To test this, we utilized the same MDA-MB-231 cell lines tested in the xenograft, to compare a constitutive shRNA-mediated knockdown using MDA-MB-231.mlp.sh*mdm2* and MDA-MB-231.mlp.sh*mdmx* as previously described [4].

**Figure 2:**
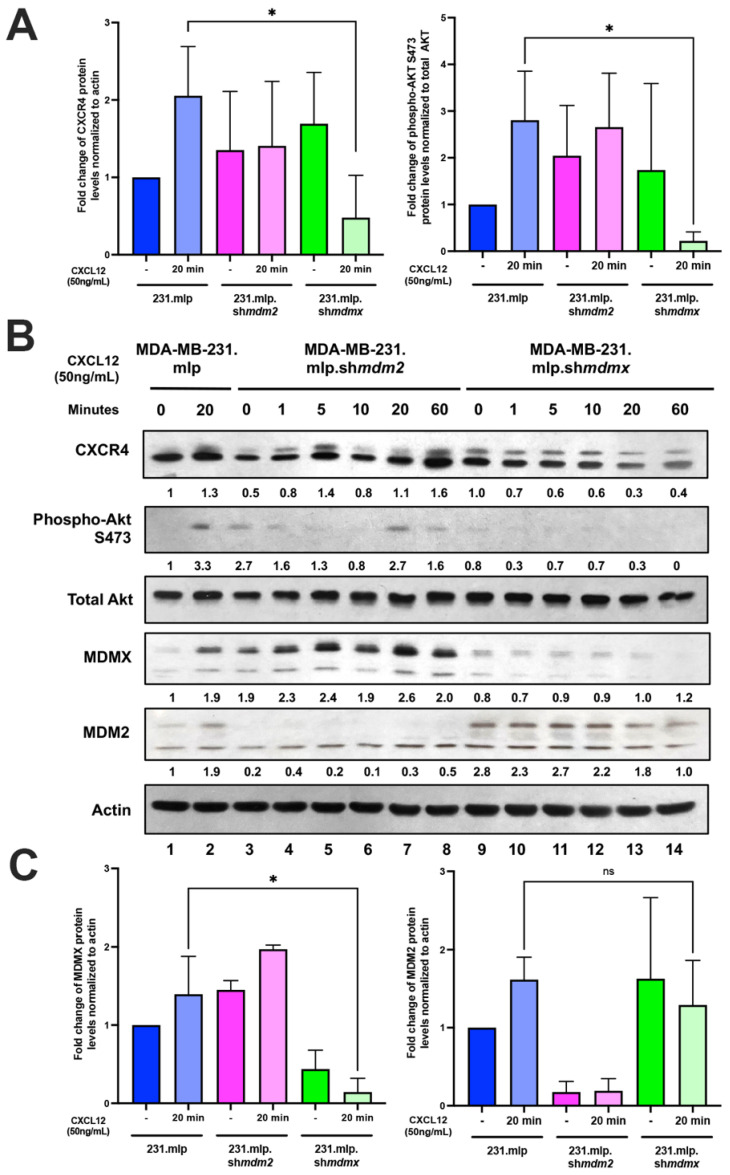
Knockdown of MDMX in MDA-MB-231 cells disrupts CXCL12 signaling to upregulate CXCR4, MDM2, and AKT activation. MDA-MB-231.mlp, MDA-MB-231.mlp.sh*mdm2*, and MDA-MB-231.mlp.sh*mdmx* cells treated in cell culture with the addition of CXCL12 at a final concentration of 50 ng/mL in cell culture for up to 60 min. (**A**) CXCR4 and phospho-AKT S473 protein levels were semi-quantified at 20 min via ImageJ relative to actin as a loading control. Protein level analysis was carried out using Western blot results using Image J and Prism software and densitometries were measured as a ratio relative to the actin band density. Fold change was calculated relative to protein levels in the untreated 231.mlp vector control cells. Error bars represent SD. * *p* < 0.05, NS = non-significant (N = 3 biological replicates). (**B**) Immunoblot analysis for CXCR4, phospho-AKT, MDMX, and MDM2 protein levels in MDA-MB-231 or knockdown cells after the addition of CXCL12. (**C**) MDMX and MDM2 protein levels were semi-quantified at 20 min post addition of CXCL12 to the cell culture. Protein levels were normalized to actin and fold change was calculated relative to untreated 231.mlp vector control cells. MDM2 or MDMX knockdown were confirmed for each respective cell line. Protein level analysis was carried out from Western blot results using Image J and Prism software and expression scores were normalized to actin. Error bars represent SD. * *p* < 0.05, NS = non-significant (N = 3 biological replicates).

We then asked how the knockdown of MDM2 or MDMX influenced CXCL12 signaling. Importantly, the addition of CXCL12 for 20 min to MDMX knockdown, but not MDM2 knockdown, cells resulted in a significant decrease in CXCR4 protein levels and phospho-AKT S473 (quantified in Figure 2A). This suggests that the increase in CXCR4 protein mediated by CXCL12 in a cell culture requires MDMX and that CXCL12 may play a role in upregulating CXCR4 expression. When CXCL12 was added for different amounts of time, the rapid and transient upregulation pattern varied greatly. However, in MDMX-knockdown cells there was a reproducibly low expression level of CXCR4 and no AKT activation (Figure 2B, lanes 9–14 see top two panels). In contrast, in MDM2-knockdown cells, CXCL12 treatment for 20 min showed increased MDMX, CXCR4, and phospho-AKT S473 (Figure 2A; lane 8). This supported the previous in vivo data which showed that, in the absence of MDMX, the expression of the CXCR4 protein in tumors in xenograft mice does not increase [4]. The reduction in CXCR4 levels observed in both MDMX-depleted primary tumors and in the cell culture systems (confirmation of the reduction in MDM2 and MDMX shown in Figure 2C) with chemokine addition suggests MDMX is a component of the CXCL12-CXCR4 pathway. These findings may provide insights into CXCR4 biology, and the different ways in which MDM2 and MDMX are involved in regulating CXCR4 receptor levels and stromal “cross-talk” through the CXCL12-CXCR4 axis to upregulate intracellular MDMX levels.

### 3.3. Reduced Levels of MDM2 or MDMX Inhibit the Ability of CXCL12 to Promote Cell Migration

**Figure 3:**
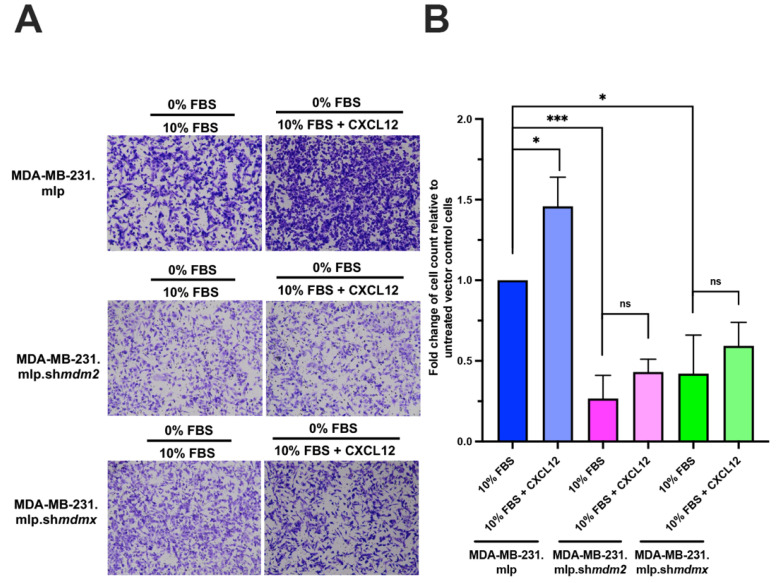
CXCL12 does not enhance chemotaxis in MDM2- or MDMX-knockdown MDA-MB-231 cells. (**A**) Representative images of crystal violet-stained cells of chemotaxis assay membrane inserts. A total of 50,000 MDA-MB-231 cells were loaded into the upper chamber in media (final vol: 200 µL). Migration was initiated by adding 500 µL of medium to the lower chamber with or without CXCL12 at a final concentration of 50 ng/mL for 24 h. Cells were incubated for 24 h at 37 degree Celsius in a 5% CO_2_ incubator. Insert was stained with crystal violet and washed with Millipore water. Representative images of stained cells on insert shown by imaging via microscopy. (**B**) Graph of % wound closure at the 24 h time point. Error bars represent SD. * *p* < 0.05, *** *p* < 0.001, NS = nonsignificant. The *p* values were calculated using two-tailed unpaired *t* tests on Prism software.

A reduction in MDM2 or MDMX decreases MDA-MB-231 cell migration and reduces CTC formation and the metastatic burden in the lungs [4]. Furthermore, the addition of CXCL12 to MDA-MB-231 cells increases their migration [8]. We therefore asked if the addition of CXCL12 enhanced migration in chemotaxis assays in a MDM2- or MDMX-dependent manner. We used a colorimetric Boyden chamber assay to assess the migration of MDA-MB-231 cells from the upper chamber of the insert through the membrane pores (Figure 3). As expected, the addition of CXCL12 to the complete media in the bottom chamber of the vector control cells significantly promoted migration (Figure 3A for representative image and 3B for quantitation). Consistent with our previous findings, the knockdown of MDM2 or MDMX significantly impeded migration compared to the vector control cells. When CXCL12 was added, the migration of the vector control cells increased (Figure 3A,B blue bars). However, both the MDM2- and MDMX-knockdown cells did not respond to the addition of CXCL12 and showed no increased migration. These results suggest that there is cross-talk between MDMX and MDM2 regarding their roles in the migration and chemotaxis of MDA-MB-231 cells and CXCL12-CXCR4-mediated cell mobility. This is in line with the previous observation of Gao et al. that both MDM2 and MDMX promote metastasis [3,4].

### 3.4. Circulating Tumor Cells Derived from Xenograft MDA-MB-231 Maintain Higher Migratory Capacity with MDM2 and MDMX but Have Reduced CXCL12 Response

**Figure 4:**
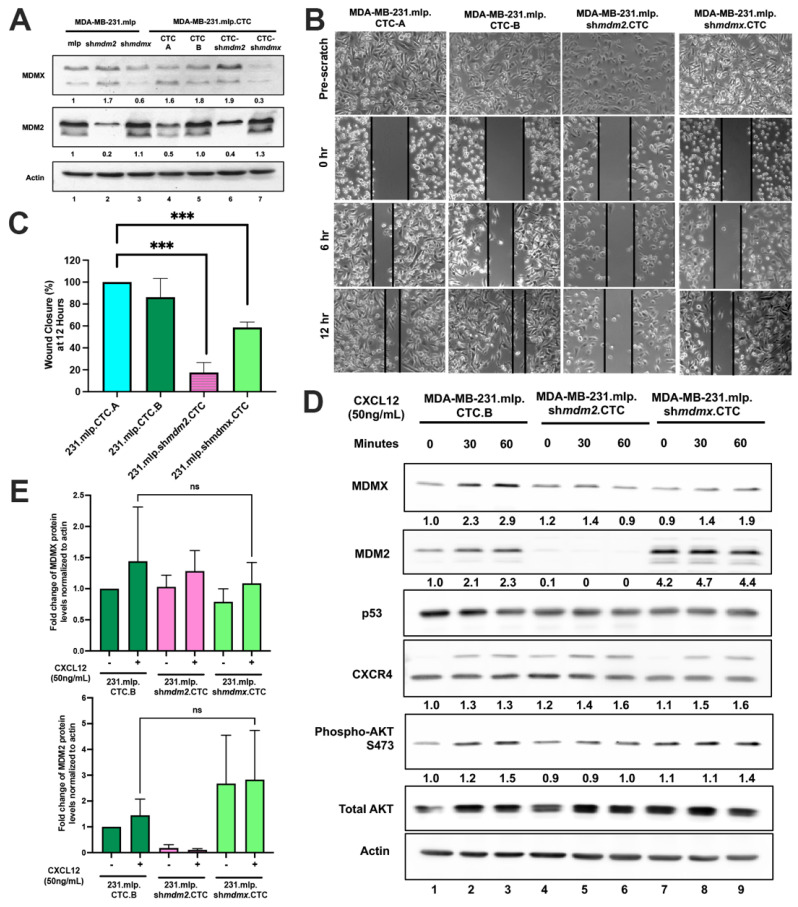
MDA-MB-231.mlp.CTC lines compared to MDA-MB-231.*shmdm2*.CTC and MDA-MB-231.*shmdmx*.CTC maintain increased migratory compacity but have reduced response to CXCL12. (**A**) Immunoblot of whole cell lysates from MDA-MB-231.mlp, MDA-MB-231.*shmdm2,* and MDA-MB-231.*shmdmx* (lanes 1–3) and MDA-MB-231.mlp.CTC A and B (lanes 4 and 5), MDA-MB-231.*shmdm2*.CTC (lane 6), and MDA-MB-231.*shmdmx*.CTC (lane 7) cell lines probed for MDMX (top) and MDM2 (middle). The loading control was actin (bottom) (**B**) Representative images of MDA-MB-231-derived CTC lines in wound healing assay. Black lines denote the borders of the scratch made. (**C**) Graph of % wound closure at 12 h time point. Error bars represent SD. *** *p* < 0.001, NS = nonsignificant. The *p* value was calculated using two-tailed unpaired *t* tests on Prism software (**D**) Immunoblot of whole cell lysates from MDA-MB-231.mlp.CTC lines treated with CXCL12 at a final concentration of 50 ng/mL for 30 and 60 min. (**E**) MDMX and MDM2 protein expression was compared using ImageJ quantitation relative to actin, respectively, as a loading control.

We observed that MDA-MB-231 cells in culture, with added CXCL12, maintained some characteristics of the primary xenograft tumor and, as such, we asked if MDA-MB-231 circulating tumor cells (CTCs) derived from mice maintain CXCL12-responsive features. It has been suggested that CXCL12 signaling is important for cells to extravasate into blood vessels and out to metastatic sites; however, the response of CTCs to CXCL12 has not been explored. MDA-MB-231 cells with either MDM2 or MDMX knockdown produce fewer CTCs and metastasis. We used our previously described protocol [4,16] to isolate the CTCs from the three MDA-MB-231 xenograft animals and found that while very few MDM2- or MDMX-knockdown cells were obtained, we were still able to establish CTC lines that we named MDA-MB-231.mlp.CTC, MDA-MB-231.*shmdm2*.CTC, and MDA-MB-231.*shmdmx*.CTC. We compared the MDMX and MDM2 protein levels in the MDA-MB-231.mlp.CTC, (Figure 4A,B) MDA-MB-231.*shmdm2*.CTC, and MDA-MB-231.*shmdmx*.CTC lines to their parental cell line (Figure 4A, compare lanes 1–3 to lanes 4–7). We observed that two MDA-MB-231.mlp.CTC lines maintained MDM2 expression (Figure 4A, lanes 4 and 5), MDA-MB-231.*shmdm2*.CTC maintained reduced MDM2 (Figure 4A, lane 6), and MDA-MB-231.*shmdmx*.CTC maintained a reduced MDMX expression (Figure 4A, lane 7). MDA-MB-231 cells were more migratory than the MDM2- or MDMX-knockdown cells when compared according to wound closure. We observed that the MDA-MB-231.mlp.CTC lines recapitulate this higher migratory feature compared to the knockdown cells (Figure 4B; quantified in Figure 4C). When we treated the CTCs in culture with CXCL12, we observed no clear change in the CXCL12-CXCR4/AKT signaling (Figure 4D,E). However, this may be due to a lack of fluid shear stress which has been shown to potentiate CTC dissemination through CXCR4-PI3K/Akt signaling [5]. We observed an upward trend of signaling in the MDA-MB-231.mlp.CTC lines but it was not statistically significant. It has been shown that tumor cells gain new characteristics when they become CTCs and future studies will be needed to address the multiple forces influencing CTC survival and extravasation.

### 3.5. CXCL12 Addition Does Not Increase Protein Half-Life Following Cycloheximide but Results in Moderately Increased Protein Levels with MG132 Inhibition of the Proteasome

**Figure 5:**
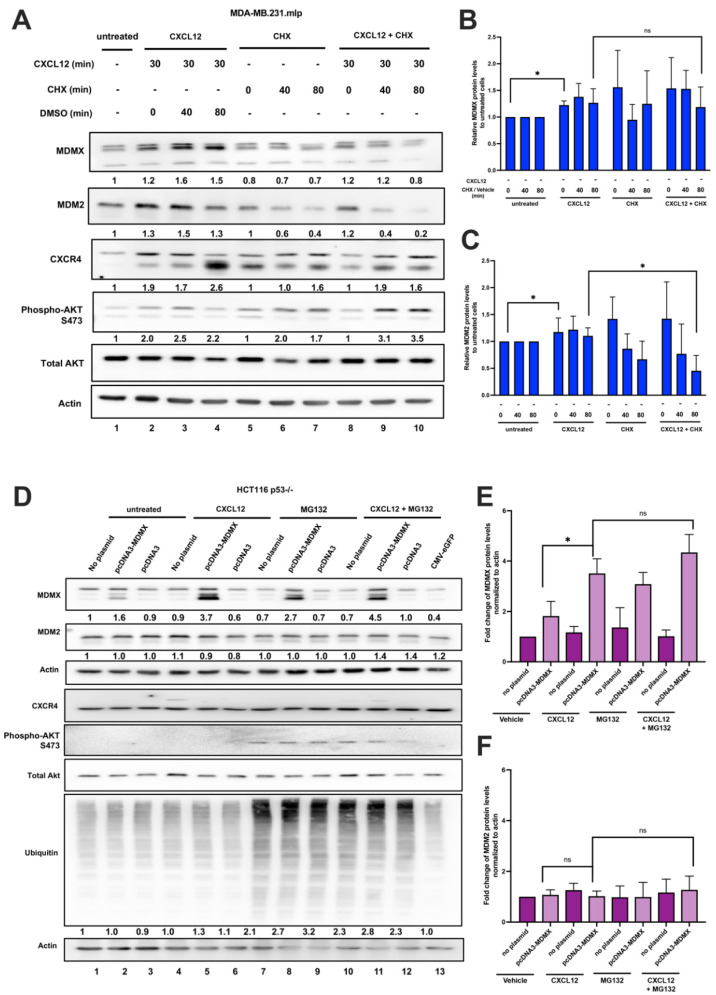
CXCL12 addition does not increase MDMX protein half-life following cycloheximide or MG132 treatment. (**A**) Immunoblot analysis of lysates from MDA-MB-231 cells treated with CXCL12 (50 ng/mL) for 30 min followed by cycloheximide (CHX) or DMSO for 40 or 80 min. Cells were harvested and lysed in CHAPS lysis buffer and subjected to immunoblotting to probe for MDM2 and MDMX. Actin was probed as a loading control. (**B**,**C**) Evaluation of Western blot band density was carried out using ImageJ and Prism software. Error bars represent SD. * *p* < 0.05, NS = non-significant. (**D**) HCT116 p53-/- cells were transfected with pcDNA3-MDMX for 24 h and then treated with CXCL12 (50 ng/mL) for 30 min followed by MG132 or DMSO for 40 or 80 min. Cells were harvested and lysed in CHAPS lysis buffer and subjected to immunoblotting to probe for MDM2, MDMX, and Ubiquitin. Actin was probed as a loading control. (**E**,**F**) Evaluation of Western blot band density was carried out using ImageJ and Prism 10 software. Error bars represent SD. * *p* < 0.05, NS = non-significant.

CXCL12 signaling in tumor microenvironments participates in signaling with an abundance of cell autonomous, and cell non-autonomous, signals working in concert. We investigated if the peptide ligand CXCL12 upregulated the MDM2 and MDMX proteins in an MDA-MB-231 cell culture by improving protein stability after synthesis (Figure 5A). To test if CXCL12 reduced MDM2 or MDMX protein turn-over, we compared cells treated with cycloheximide (CHX) to those treated with DMSO for either 40 or 80 min. When CHX was added, we observed a rapid decrease in MDM2, whereas MDMX demonstrated more stability and a slower degradation. CHX addition following CXCL12 treatment did not increase MDM2 or MDMX protein levels (Figure 5A; quantified in Figure 5B,C). As both MDM2 and MDMX are degraded by the proteasome, this suggested that CXCL12 might work in conjunction with the proteasome pathway. Because CXCL12 signaling is rapid and transient, we found it challenging to carry out the combination of CXCL12 plus MG132 proteasome inhibitor in the MDA-MB-231 cells. In order to address signaling in the absence of wild-type p53, in a no-p53-expression cell line, we shifted to using the exogenous expression of MDMX in HCT116 p53-/- cells transfected with pcDNA3-MDMX. This showed significantly increased the levels of MDMX protein by 3-fold (Figure 5D; and quantified in Figure 5E,F). Herein we observed that the addition of CXCL12 to p53-null colorectal cancer cells increased the levels of exogenously expressed MDMX. However, no increase in the endogenous MDM2 was observed. We recognize that the signaling in HCT 116 p53-/- cells is very different from that in MDA-MB-231 breast cancer cells. However, to find out if the increase in protein levels in response to CXCL12 addition was due to a reduction in proteasomal degradation, we added MG132 or DMSO to the cell culture to block the proteasomal degradation without or after adding CXCL12. The addition of MG132 to the cells increased the total ubiquitinated protein levels and MDMX levels by 3 fold. We observed a slight, but not statistically significant, increase in MDMX protein levels when CXCL12 was added to the cells followed by MG132. This suggested that the CXCL12 pathway can promote the stability of the MDMX protein.

## 4. Discussion

In this study, we investigated the cross-talk between the CXCL12/CXCR4 axis and MDM2/MDMX in the context of breast cancer cells that express mutant p53 protein. This is important because mutant p53, and not wild-type p53, is expressed in 80% of triple-negative breast cancers (TNBCs). Our previous studies showed a positive correlation between CXCR4 expression in TNBC xenograft tumors, and when cells were grown outside the stromal environment this was not observed [4]. This suggested that a stromal cell-derived factor was responsible, and a likely candidate was CXCL12. Herein, we showed that the exogenous addition of the CXCL12 peptide, in some cellular settings, upregulated not only the previously documented activation of CXCR4 and kinase signaling, but also led to the upregulation of MDM2 and MDMX protein levels. In MDA-MB-231 cells, the upregulation of both pathways required MDMX which suggested a feed-forward loop. Here, the G-protein-coupled receptor activated a pathway to upregulate MDM2 and MDMX, and then in the setting of mtp53, this further upregulated MDM2, MDMX, CXCR4, and AKT signaling. Moreover, the positive regulation dependent on MDMX was supported by the fact that when MDMX was depleted the CXCL12-mediated increase in cell migration was reduced. Additionally, the addition of CXCL12 to HCT116 p53-/- cells increased the level of exogenously expressed MDMX. MDMX and MDM2 work together to allow MDM2 to function as a potent E3 ubiquitin ligase that can target wild-type p53 and MDM2 and MDMX for degradation. In a wild-type p53 setting, this functions to decrease the p53-mediated activation of apoptosis and cell cycle arrest. However, in an mtp53 context the MDM2/MDMX axis also works to promote metastasis. The ability of CXCL12 to upregulate MDM2 and MDMX has the potential to promote alternative transformative properties dependent on cell context and CXCR4 expression levels.

The results presented herein show a positive feed-forward pathway between CXCL12-CXCR4 and increased MDM2 and MDMX protein levels through a mechanism that is not transcriptional. The addition of CXCL12 was unable to increase MDMX protein stability after cycloheximide treatment but augmented the increase in MDMX protein achieved by the inhibition of the proteasomal degradation.

It was not surprising that in all our experiments the knockdown of MDM2 resulted in an increase in the MDMX protein. This is because MDMX is an E3 ubiquitin target of MDM2, and therefore, a reduction in MDM2 causes decreased MDMX degradation. What was surprising was that the addition of CXCL12 reproducibly caused an increase in MDM2 and MDMX protein levels in MDA-MD-231 cells. The CXCL12 peptide ligand is known to increase the levels of a number of proteins including CXCR4. The question remains why, in the xenograft tumor model, the MDMX-associated upregulation of CXCR4 was a result of an increase in mRNA and protein, and why, in this exogenous model of the addition of CXCL12, the only increase observed was in the CXCR4 protein levels.

**Figure 6:**
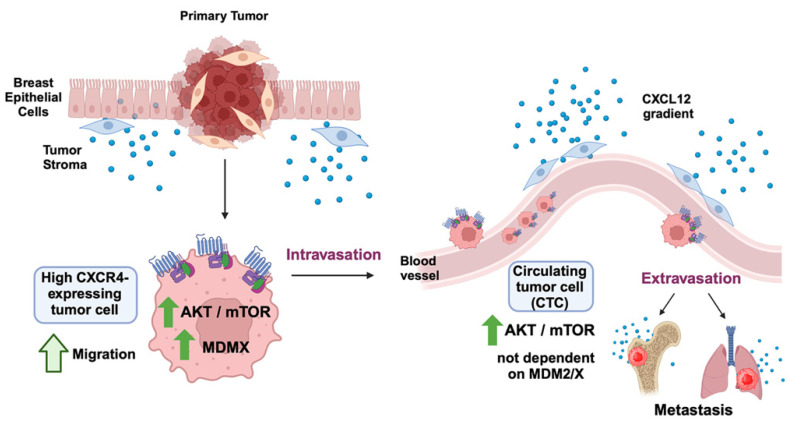
A model of CXCL12/CXCR4/AKT signaling to the MDMX/MDM2 axis. CXCL12/CXCR4/AKT signaling works to upregulate MDMX in the primary tumor in cooperation with the TME and results in a feed forward activation loop. This promotes intravasation of the cancer cells into the blood stream and the promotion of CTCs. The CTCs are then able to survive but have a reduced CXCL12 feed-forward loop with MDMX and MDM2 which we posit is re-established when cells extravasate into CXCL12-rich environments to form metastasis.

The TME produces a number of different factors that promote metastasis. These include the well-documented stromal-derived factor CXCL12 as well as inflammatory cytokines. As such, the possibility exists that many cell non-autonomous factors influence CXCR4 upregulation in the TME, and that these factors are absent in the experimental setting of the cell culture dish. The CXCL12/CXCR4 axis is currently being explored as a target for blocking metastasis. The antagonist AMD3100 has the potential to be used as a drug to block metastasis. Future work should address if CXCR4 inhibitors can reduce the expression of MDM2 and MDMX and AKT signaling activation. Our data demonstrate that the primary tumor cells have a stronger CXCL12/CXCR4 signaling axis than the CTCs. As such, the AMD3100 inhibitor (which blocks CXCR4) may work well to block primary tumor cells from intravasation into the blood stream. The CTCs may have additional signaling pathways in place for promoting extravasation into CXCL12-rich environments. When extravasation of CTCs occurs and metastases are formed, the MDM2/MDMX’s cross-talk with the CXCL12/CXCR40-positive loop may become re-established to fuel further metastasis. Dual blocking of the two pathways (modeled in Figure 6) is a potential way to target the primary tumor and the metastatic lesions to block further metastasis. The compound ALRN has the ability to block both MDM2 and MDMX but clinical trials with the drug demonstrated profound side effects that may be due to activation of the wild-type p53 pathway [28]. Future studies will be needed to determine the best practice and compounds for the simultaneous inhibition of the CXCL12/CXCR4 axis and the MDM2/MDMX axis to block breast cancer metastasis. Until that time, the CXCL12/CXCR4/MDM2/MDMX axis in tumor metastasis is speculative since only the role of in vitro cell migration has been assessed in the current manuscript. However, it is determined that the inhibition of CXCR4 expression is a target for blocking TNBC metastasis [29]. In fact, radiopharmaceutical therapy against fibroblast-activated proteins in combination with the CXCR4 antagonist AMD3100 suggests it is a potential treatment against TNBC with limited side effects [30]. As such, it will be interesting in the future to determine if a similar combination therapy against MDMX with the CXCR4 agonist may be an effective treatment for TNBC.

## 5. Conclusions

We demonstrated a positive feed-forward connection between the CXCL12/CXCR4 signaling axis and the MDM2/MDMX-mtp53 oncogenic pathways to promote cancer cell migration and metastasis (model shown in Figure 6). This is the first identification of this regulatory loop, suggesting the pathways work together to potentiate metastatic progression from the primary tumor. Our study revealed an important role for MDMX in CXCL12 signaling for kinase activation and chemotaxis. Furthermore, we observed that CXCL12 not only increased the level of endogenous MDMX protein but was also able to increase the level of HCT1116 p53-/- exogenously expressed MDMX protein. This indicates that cell non-autonomous CXCL12 expression in the stroma may be a TME extracellular “cross-talk” signaling factor that promotes metastasis of cells from primary tumors by upregulating tumor-associated cell autonomous MDMX expression. Our results suggest that the upregulation of MDMX expression may be a result of a CXCL12-mediated increase in MDMX upstream of the proteasomal degradation pathway. This is the first work to identify a feed-forward regulatory loop between the extracellular signaling axis CXCL12/CXCR4 to oncoprotein MDMX, which increases MDMX and promotes metastasis.

## Data Availability

The original contributions presented in the study are included in the article/Supplementary Materials, further inquiries can be directed to the corresponding author.

## Supplementary Materials

The following supporting information can be downloaded at: https://www.mdpi.com/article/10.3390/cancers16244194/s1, Supplemental Figure S1. CXCL12-CXCR4/AKT signaling increases MDM2 and MDMX protein in different mtp53-expressing cell lines; Supplemental Figure S2. CXCL12 ligand addition into cell culture does not increase MDMX or MDM2 mRNA expression; Supplemental Figure S3. mRNA expression of exogenous addition of CXCL12 ligand to MDA-MB-231-derived CTC cells in cell culture.
