# Supplementary material for: Chemokine CXCL12 Activates CXC Receptor 4 Metastasis Signaling Through the Upregulation of a CXCL12/CXCR4/MDMX (MDM4) Axis
Source: Cancers (Basel). 2024 Dec 16;16(24):4194. doi: 10.3390/cancers16244194 (PMC11674518; doi:10.3390/cancers16244194)
Supplement: Supplementary file 1 [file cancers-16-04194-s001.zip › cancers-3295280-supplementary.pdf]

SUPPLEMENTAL FIGURES

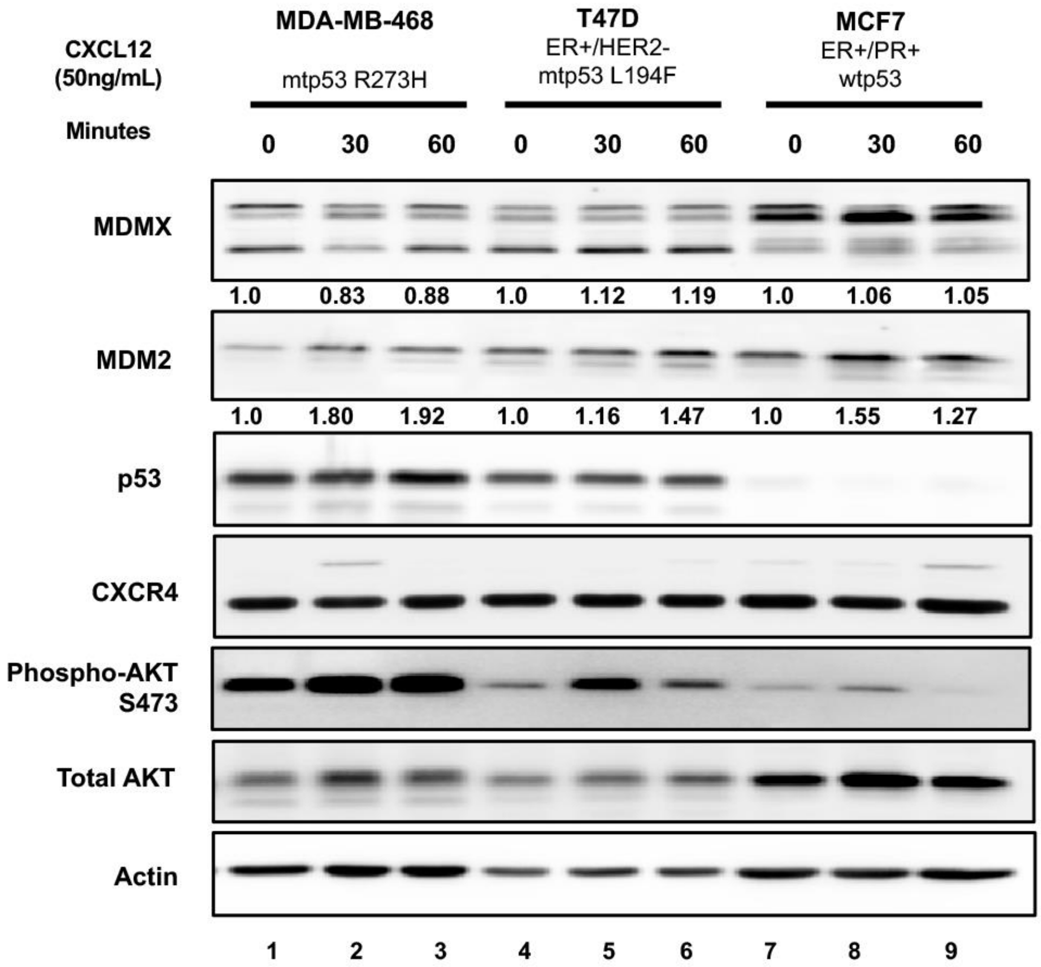

Supplemental Figure S1. CXCL12-CXCR4/AKT signaling increases MDM2 and MDMX protein in different mtp53-expressing cell lines. Immunoblot of whole cell extracts from mtp53-expressing cell lines, MDA-MB-231, MDA-MB-486, T47D cells, and wtp53-expressing cell line, MCF7, probed for MDM2 and MDMX. Quantification of MDM2 and MDMX in three biological replicate experiments.

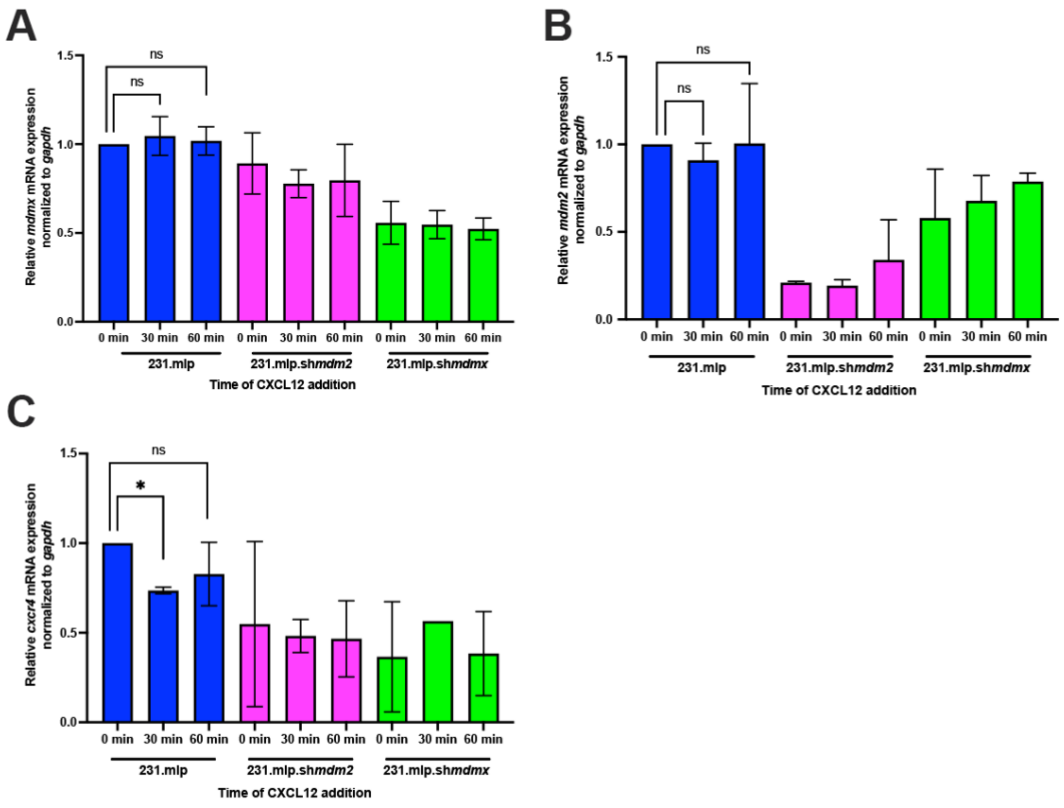

Supplemental Figure S2. CXCL12 ligand addition into cell culture does not increase MDMX or MDM2 mRNA expression. (**A**-**C**) Total RNA was extracted from MDA-MB-231.mlp and their respective MDM2 or MDMX knockdown cell lines. cDNA was synthesized and RT-qPCR was performed using TaqMan probes specific for (**A**) *MDMX*, (**B**) *MDM2*, (**C**) *CXCR4*, respectively. Expression analysis was done using Prism software and expression scores were normalized to *GAPDH*. Error bars represent SD. * $p < 0.05$, NS = non-significant (N = 3 biological replicates).

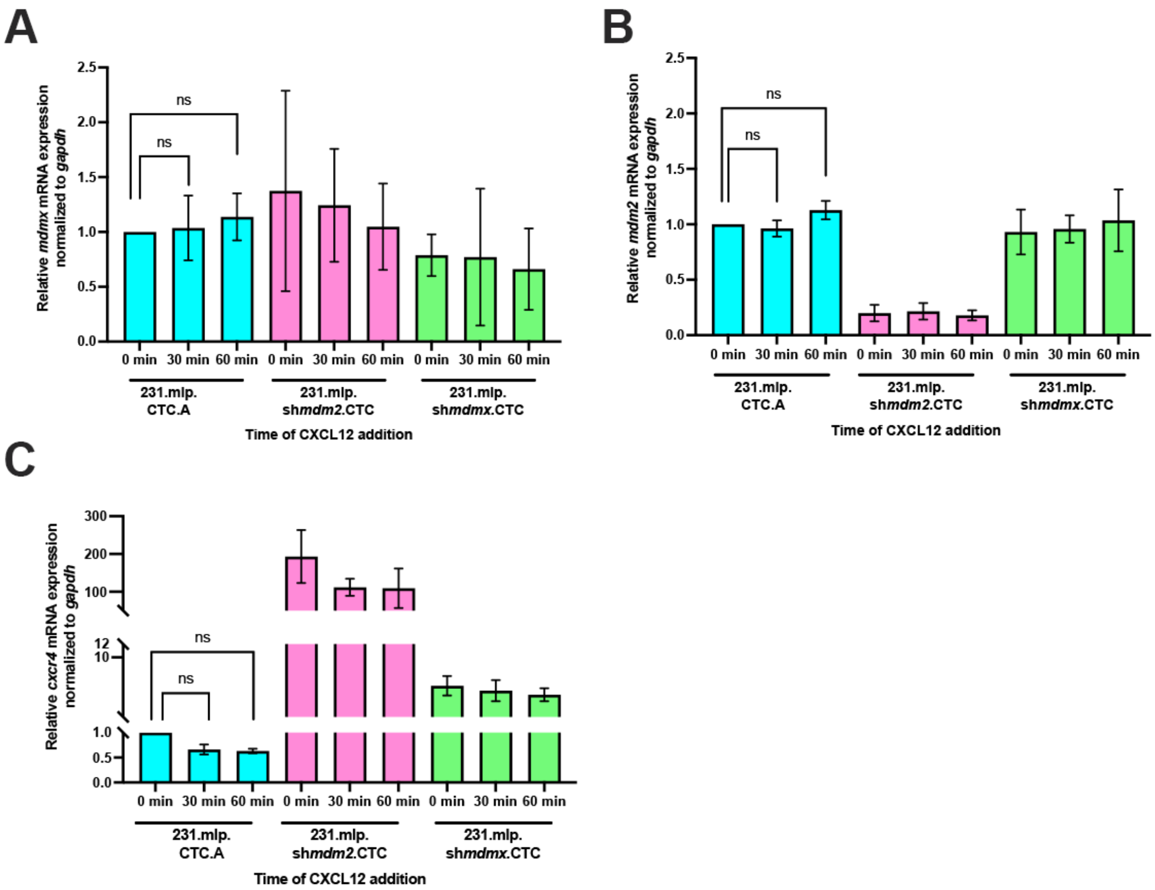

Supplemental Figure S3. mRNA expression of exogenous addition of CXCL12 ligand to MDA-MB-231-derived CTC cells in cell culture. (**A**-**C**) RT-qPCR of MDA-MB-231-mlp.CTC, MDA-MB-231.mlp.sh*mdm2.*CTC, and MDA- MB-231.mlp.sh*mdmx*.CTC cells. Expression analysis was done using Prism software and expression scores were normalized to *GAPDH*. Error bars represent SD. NS = non-significant. (N = 3 biological replicates).
