# Supplementary figures and images for: Chemokine CXCL12 Activates CXC Receptor 4 Metastasis Signaling Through the Upregulation of a CXCL12/CXCR4/MDMX (MDM4) Axis
Source: Cancers (Basel). 2024 Dec 16;16(24):4194. doi: 10.3390/cancers16244194 (PMC11674518; doi:10.3390/cancers16244194)

**Figure 1**

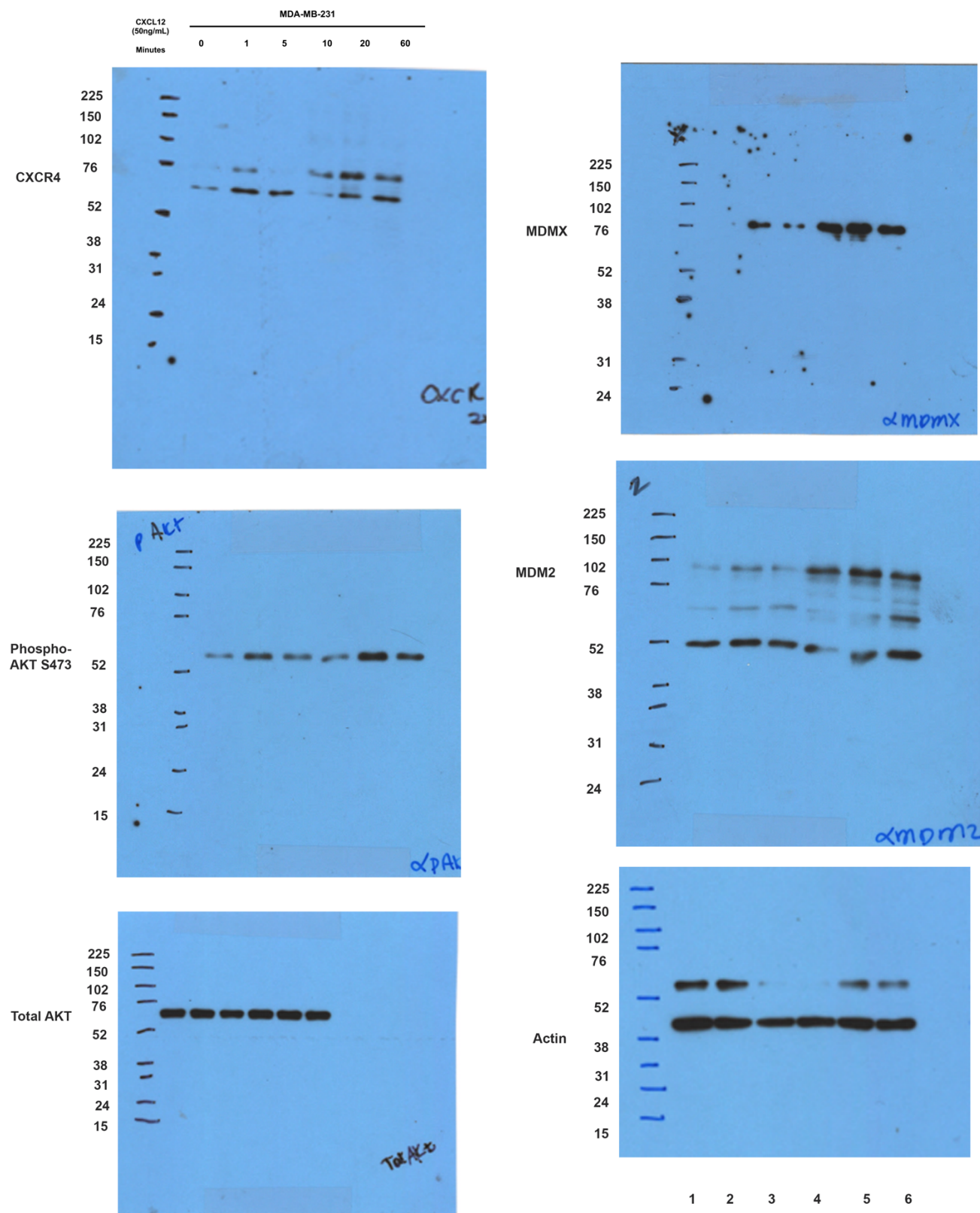

Figure 2

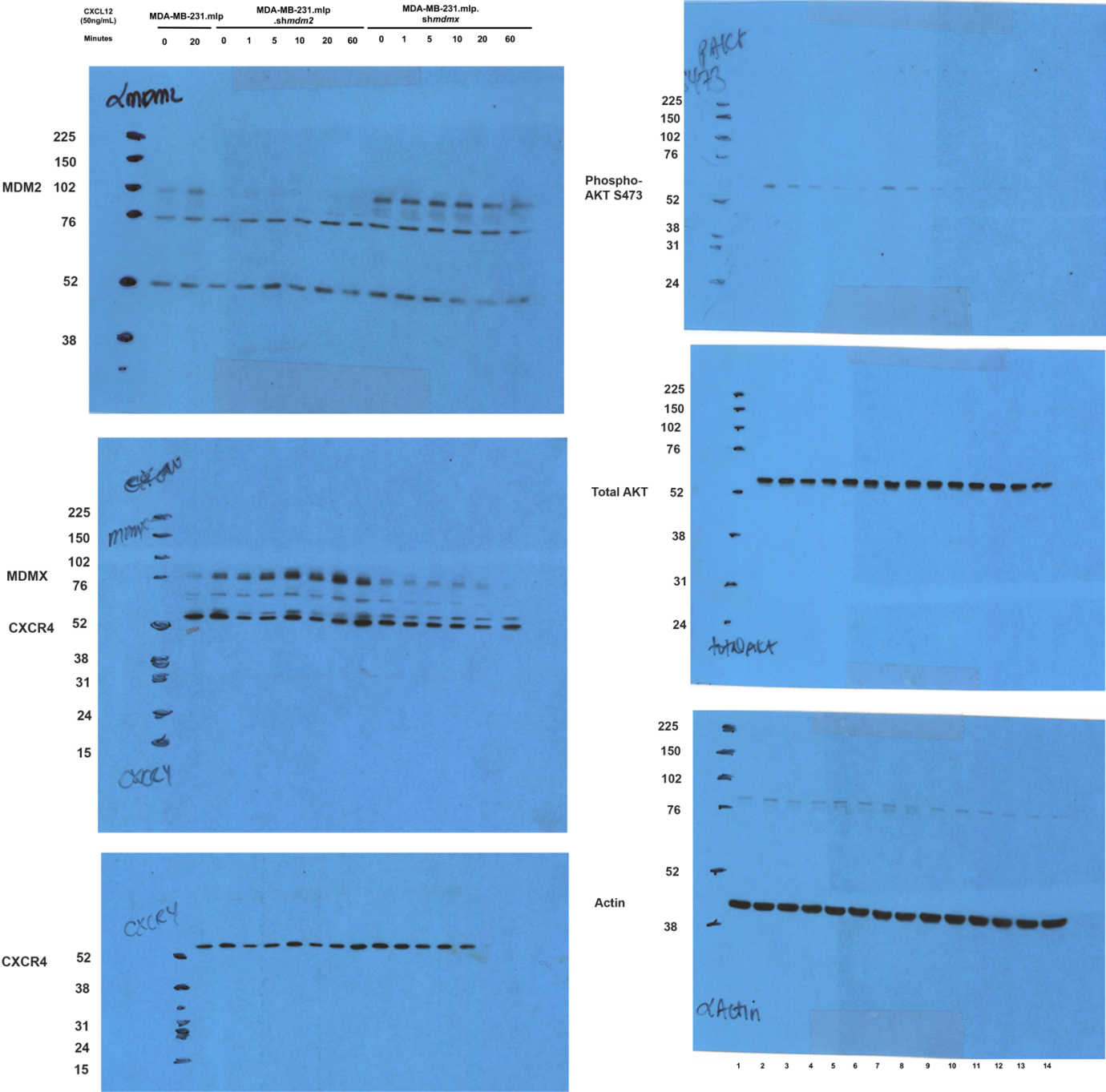

Figure 4A

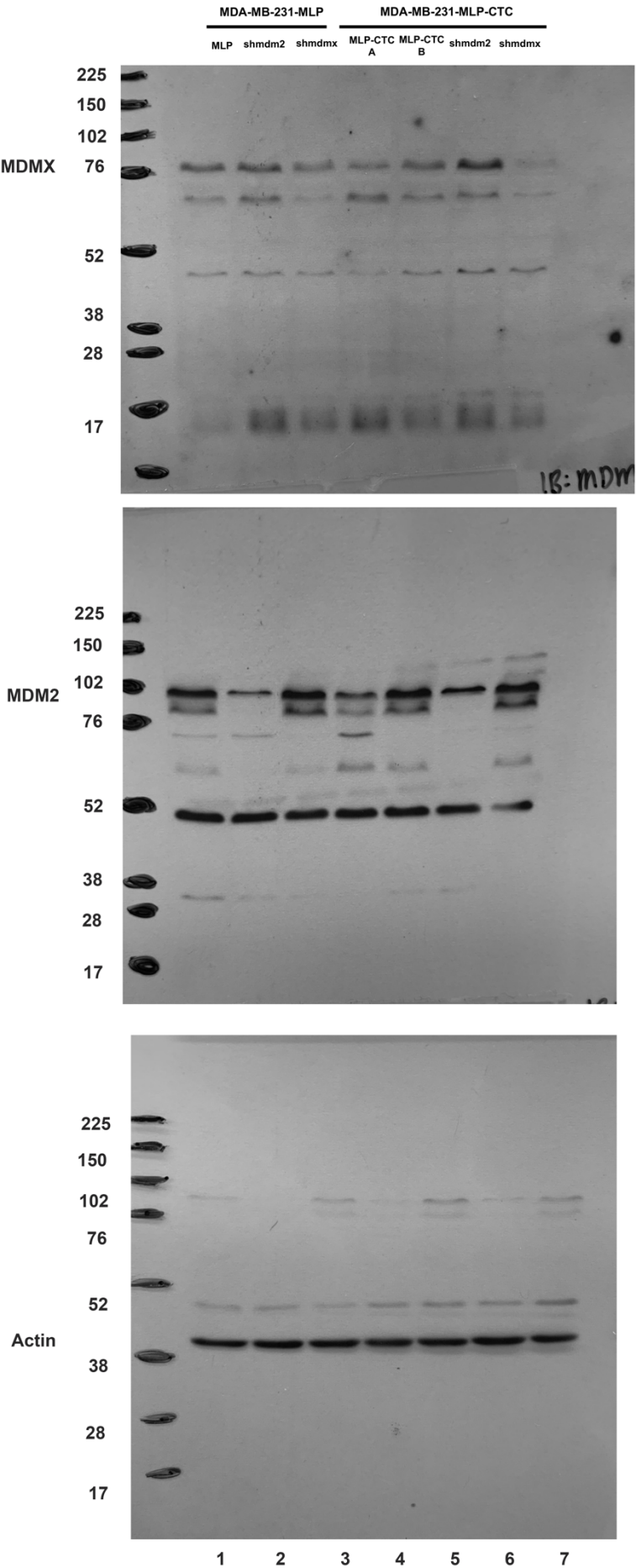

Figure 4D

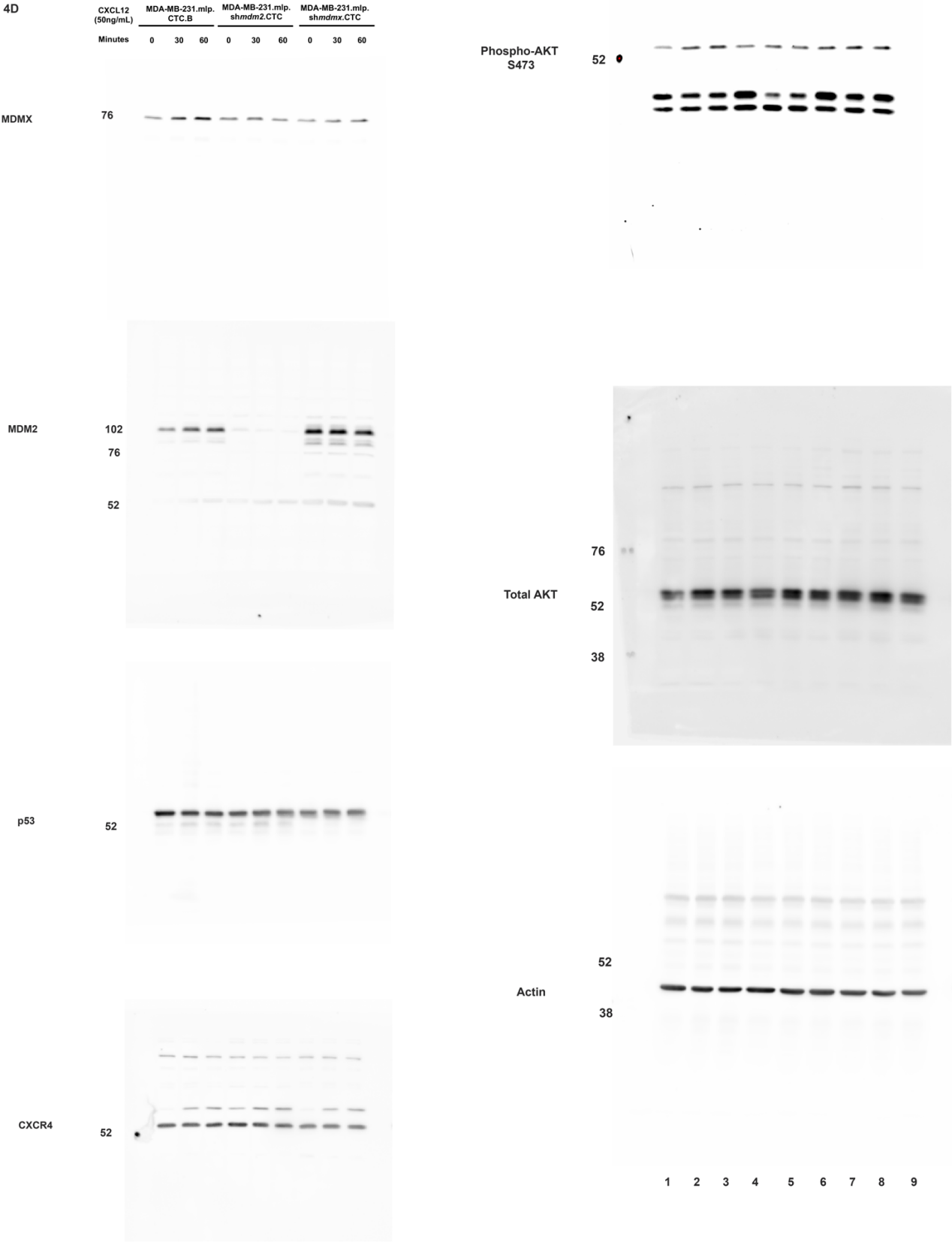

Figure 5A

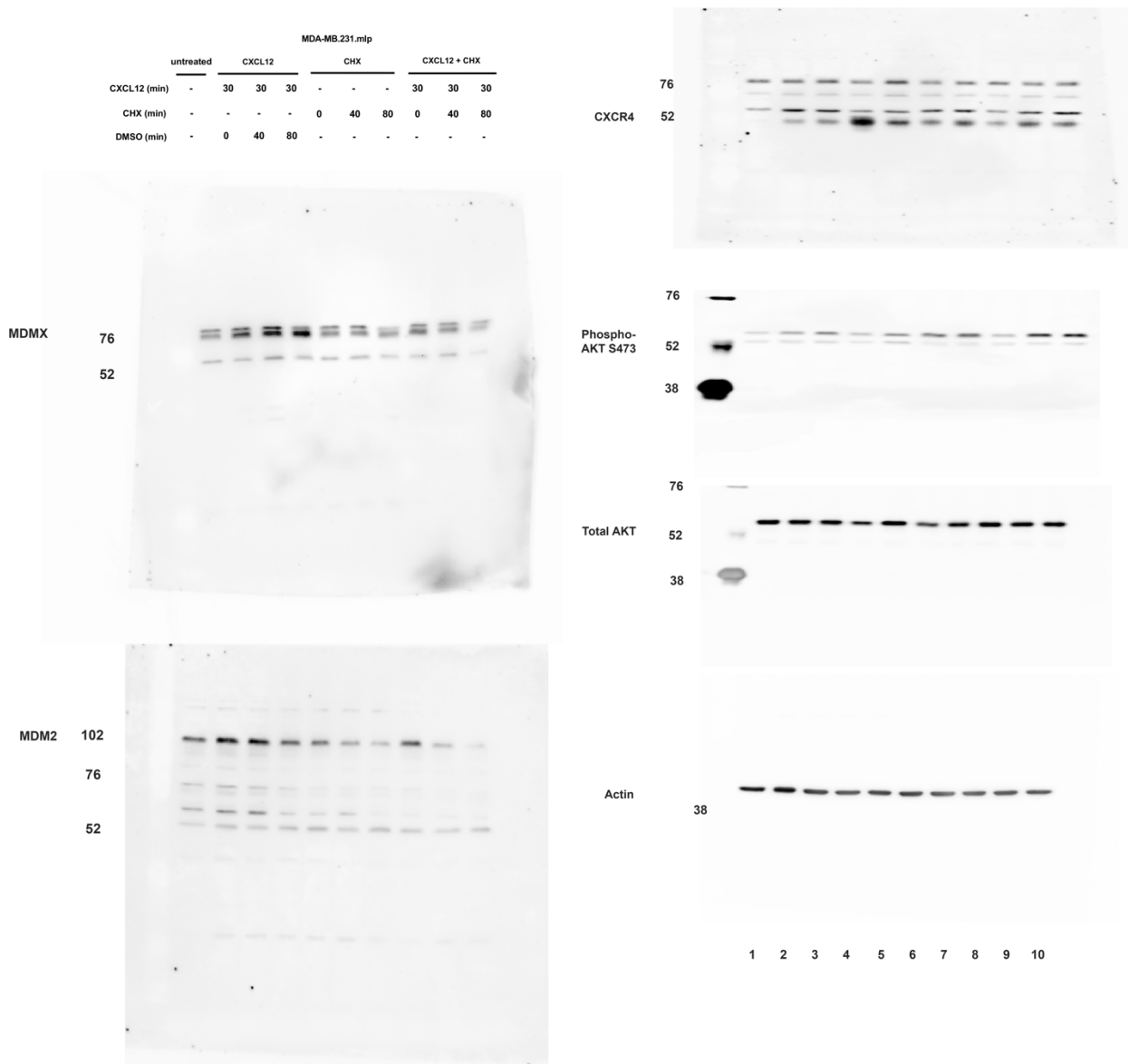

Figure 5D

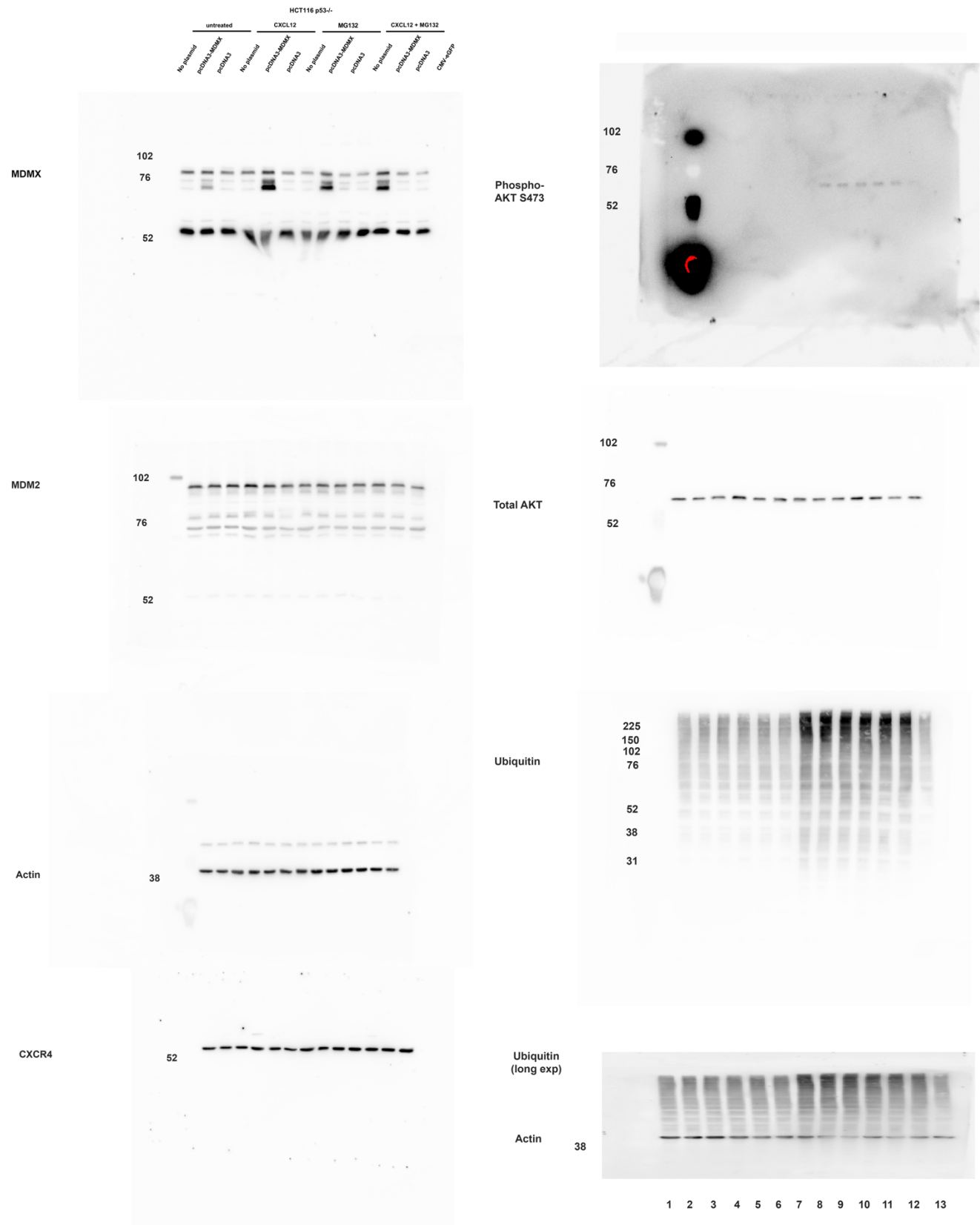

Supplemental Figure 1

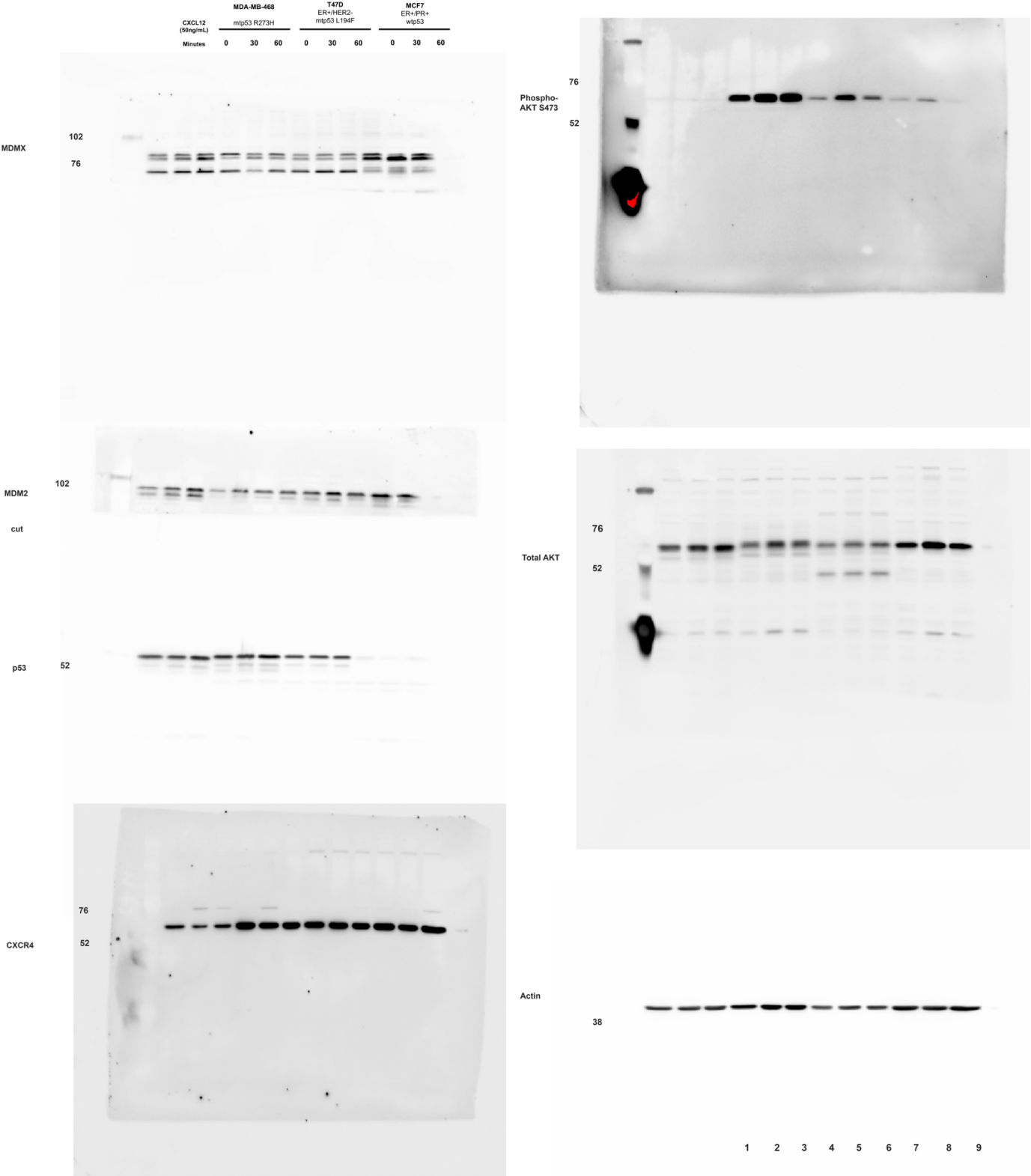

Supplement: Supplementary file 1 [file cancers-16-04194-s001.zip › Full Membranes.pdf]
